# Performance evaluation of Rayto RT-7600Vet hematology analyzer in side-by-side comparison with manual hematological methods for apparently healthy Cholistani cattle blood

**DOI:** 10.1371/journal.pone.0302617

**Published:** 2025-03-11

**Authors:** Umer Farooq, Mushtaq Hussain Lashari, Zia Ur Rehman, Musadiq Idris, Haroon Rashid, Shagufta Nasreen, Farah Laraib, Rubaisha Ameer, Maryam Chauhdary, Iram Fatima

**Affiliations:** 1 Department of Physiology, The Islamia University of Bahawalpur, Bahawalpur, Pakistan; 2 Department of Zoology, The Islamia University of Bahawalpur, Bahawalpur, Pakistan; The Ohio State University, UNITED STATES OF AMERICA

## Abstract

The present study is the first from Pakistan being reported with an objective to assess performance of Rayto RT-7600Vet hematology analyzer (HA) for Cholistani cattle blood (n = 134), in comparison to the manual hematological methods. The four hematological attributes *viz*. total erythrocyte count (TEC), hemoglobin (Hb), packed cell volume (PCV) and platelet count (PLT) were deduced through HA (A) and manual (M) methods. Various statistical tests were implied to ascertain level of interrelationship, accuracy and level of agreement between the two methods. All attributes attained through manual methods had high positive, leptokurtic distribution (having many outliers) except for PLT-M and PCV-M. The coefficient of variation for attributes attained through HA and manual methods ranged from 16–24% and from 16–59%, respectively. Comparison between the overall results revealed that all the studied attributes, except TEC, were significantly (P≤0.05) different for both methods. A weak relationship was noticed between the attributes attained through two methods as indicated by weak r-values and adjusted r-square values. The reliability level of estimating Hb and PCV had highest intraclass correlation coefficient value of 0.722 and 0.555 for average measures, respectively. However, accuracy level, as determined through Lin’s concordance correlation coefficient was highest for TEC (0.9504) for both analytical methods. Poor level of agreement, in general, was shown for the two methods of analysis regarding all four hematological attributes through Bland and Altman test. In conclusion, the Rayto RT-7600Vet) may present data having higher skewness, kurtosis, and CV%, however, they are valid for multi-species hematological analysis. Caution must however, be taken in interpreting their results with corrected reference intervals and CV% for each machine and for each tested attribute.

## Introduction

In the developing and under-developed countries of the world (including Pakistan), diagnosis/prognosis of various pathological conditions is normally made through physical examination allied with a thorough anamnesis, both in human and veterinary medical practice. Since long, human medical practice has surpassed veterinary medical practice in the use of reliable, quick, bed-side, point-of-care tests (POCTs) and instruments for definitive diagnosis/prognosis. Such instruments mostly include serum chemistry analyzers and automated hematology analyzers (HAs) [1, 2]. Complete blood count (cbc), attained through these HAs, is of prime importance for every physician, as it provides a deeper insight into the individual’s health and abnormality. Resultantly, the automated HAs are considered pivotal for every human clinical laboratory and health facility, globally. Veterinary hematology, on the footsteps of human hematology, has made substantial advancements in the last decade or so. And automated multi-species HAs are frequently being used for diagnosis/prognosis of various blood-borne disorders in different species throughout the world [3–5].

Since their emergence as Coulter Counters (based upon principle of electrical impedance), the HAs have seen technological advancements [6, 7]. At the moment, 3-part and 5-part hematology analyzers are in vogue. By 1980s, a HA could produce more than 10 blood attributes at a fast speed and with substantial precision. These instruments have reduced the time of analysis of a blood sample from 45 minutes (through manual hematological methods) to a mere 15 second [7]. Though they have uncountable advantages, yet their expensiveness, periodic maintenance, timely validation, expensive reagents, and need of skillful personnel to use them are a few factors which hinder their use especially in the resource-poor settings. Examination of a peripheral blood slide (PBS), therefore, still remains an inexpensive, field-oriented and quick method for determining blood cell morphology, and it cannot be considered as a ‘lost art’.

The fast-paced technological advancement in the field of hematology and launch of current products are the two main driving factors for the exponential growth in global HA and reagents market. In 2021, the global HA and reagents market size was valued to be 2.04 billion USD with an expected value of 3.90 billion USD by 2030 (an increase at a compound annual growth rate of 7%) [8]. Sysmex Corporation, Japan (with its facilities and centers throughout the world) is considered to be the leading global firm for devising HAs. However, other countries such as USA, France, Germany and China have also initiation their production of HAs. Amongst these, the Chinese machines are the cheapest and are widely being purchased by poorer countries such as Pakistan, India, Singapore, and Middle Eastern countries [9]. The Rayto Life and Analytical Science Co. Ltd., China is a leading Chinese firm which is manufacturing and marketing the HAs both for human and veterinary medical practice to various countries of the world with its major market in underdeveloped/under-developing countries [10–13]. It has recently given its sole dealership to a company of Pakistan dealing in science equipment. And sale of both human and veterinary HAs by this company has substantially escalated in the last 5 years or so [14].

Considering the vitality of a cbc report, it is inevitable that the results provided by the hematology analyzers must be valid and reliable, in order to approach a definitive treatment. It is hence required that these machines may periodically be validated, quality control to be kept strict, and their performance may be evaluated [5, 15, 16]. Extensive studies have globally been reported regarding the validation and performance of various veterinary hematology analyzers [11, 17, 18]. To date, there is no such study which may have evaluated the clinical performance of such multi-species HAs in Pakistan. The present study is hence, the first report of its kind from Pakistan regarding the performance evaluation of a multi-species HA Rayto RT-7600Vet in comparison to manual hematological methods for the blood of apparently healthy Cholistani cattle. This study included four main hematological attributes *viz*. total erythrocyte count (TEC), hemoglobin (Hb), packed cell volume (PCV) and platelet count (PLT) which normally make major component of a cbc report for diagnostic/prognostic purposes.

## Materials and methods

### Geo-location of the study

The present study was simultaneously carried out at the Cholistan desert, Pakistan and the post-graduate (PG) laboratory of the Department of Physiology, The Islamia University of Bahawalpur (IUB), Pakistan. The desert is located at latitudes 27°42´ and 29°45´North and longitudes 69°52´ and 75°24 east, with an elevation of 112 meters above sea level. This area has a monsoonal, hot, dry subtropical climate with an average annual rainfall of 180 mm. With a daily high that regularly hits 45°C, June is the hottest month of the year, with an average annual temperature of 28.33°C [19]. The ecological, environmental and livestock-associated lifestyles of the desert nomads have been reviewed earlier [19–21].

### Study animals

Regardless of their age or sex, Cholistani cattle (n = 134) were chosen at random for blood sampling from pastoralists who live on the move. All the animals were raised in pastoral livestock production system, whether transhumanistic or nomadic, with similar management and feeding practices. Pastoralists typically practice split-herding for their livestock, whereby the juvenile animals are kept in pens close to "Tobas," which are man-made or naturally occurring desert water reservoirs, and the adult animals are sent out to graze until dusk [19, 22]. The overall well-being of the animals was determined by means of a comprehensive questionnaire administered to the livestock owners and clinical indicators. The study did not include the animals whose anamnesis from the pastoralist herders showed that they were lethargic, depressed, off-feed, and separated from the herd.

### Ethics statement

The research work was approved by the Departmental Research Ethics Committee, Department of Physiology, IUB, Pakistan vide Letter No PHYSIO-77/2024-111 dated 13-01-2024.

### Blood collection

For blood sampling, the study animals were restrained in the field with the help of the owners of the livestock in accordance with the state of the field and the workplace. Using a disposable syringe, blood was aseptically drawn from the coccygeal vein. A 5mL sample of each animal was obtained aseptically in a purple-topped vacutainer (Becton Dickinson, USA) containing EDTA as an anticoagulant. Each animal was bled once, with the same personnel, and at the same time, in order to minimize stress. After being gently inverted, the blood samples were chilled and sent in an ice-box to the PG Laboratory, IUB, Pakistan. Hematological analyses were carried out within 4hrs, preferably by the same person.

### Hematological analyses

- **Through hematology analyzer**: Automated veterinary HA (Rayto RT-7600Vet, China) was used to analyze blood samples for various hematological attributes *viz*. total erythrocytic count (TEC-A), hemoglobin (Hb-A), packed cell volume (PCV-A), mean corpuscular volume (MCV-A), mean corpuscular hemoglobin (MCH-A), mean corpuscular hemoglobin concentration (MCHC-A) and platelet count (PLT-A). The Rayto RT-7600Vet (China) is a 3-part (lymphocytes, intermediate cells and granulocytes), multi-species hematology analyzer meant for the blood analysis of cats, dogs, rabbits, pigs, horses, goats, monkeys, and four other self-defined animals. It uses the principle of impedance for counting and differentiating blood cells and principle of colorimetry for Hb determination. This analyzer provides 23 test items/blood analysis attributes including three histograms each for white blood cells (WBCs), TEC and PLT. The technical characteristics of the analyzer include a minimum whole blood volume of 9.8μL, pre-diluted blood volume of 20μL, test rate of about 1 minute/essay, and a working environment of 15-35°C with humidity of 10–90%. It uses lyse solution, cleanser and a diluent for measurements and maintenance. As per its instructions manual, accuracy of test results is attained by using the reagents provided along with the instrument. The blank tests only show results for WBCs, TEC, PCV, Hb and PLT as per the reference ranges of ≤0.2×10^9^/L, ≤0.02×10^12^/L, ≤1g/L, ≤0.5% and ≤10×10^9^/L, respectively. The settings for reference intervals of each species can be set manually. Values in the cbc report which are outside the normal range are marked automatically in the report. The quality control (QC) of the analyzer includes two methods namely L-J QC and R-X QC [23].
- **Through manual methods**: Erythrocytic count (TEC-M) and platelet count (PLT-M) was carried out with an upgraded Neubauer counting chamber. The TEC-M counting was conducted on blood diluted with Hayem’s solution (SDL Scientific Enterprise, Pakistan) (1:200) [24]. Similarly, platelet count (PLT-M) was done with blood diluted with Rees and Ecker solution (Ricca Chemical Company, USA) (1:200) [25]. Counting was conducted by two trained personnel and mean values were taken in order to eliminate interpersonal error. The hemoglobin (Hb-M) was determined using Drabkin’s reagent (SDL Scientific Enterprise, Pakistan) with gold-standard cyanmethemoglobin method and commercial kit (AMP Diagnostics, BD6100E, Ameda Labordiagnostik GmbH, Germany) [26]. For packed cell volume (PCV-M), the gold-standard microcentrifuge method was utilized according to which the blood-filled capillary tubes were centrifuged in a microcentrifuge (Sigma Aldrich, Model 5254, Germany). And reading was taken as percentage with the help of a card reader [27]. The MCH-M, MCV-M and MCHC-M were calculated using prescribed formulae [28].

### Statistical analyses

Statistical Package for Social Sciences (Windows Version 12, SPSS Inc, Chicago, IL, USA) was used for data analyses. Normality of the data and homogeneity of variance were tested through Shapiro-wilk test and Levene’s test, respectively. For the purpose of analyses, data was grouped as per age (young, n = 47; adult, n = 87) and sex (males, n = 38; females, n = 96). Difference between two analytical methods *i*.*e*. through hematology analyzer and manual methods for overall as well as for group-wise data was attained through independent t-test. Pearson’s correlation coefficient and linear regression were implied to assess the level of relation between the studied attributes attained through two analytical methods, and to deduce the regression prediction equations, respectively, and is shown in scatterplots. Three tests were implied to check the level of agreement between the two analytical methods *viz*. Bland and Altman, Cronbach Alpha and Intraclass Correlation Coefficient (ICC). In order to ascertain reliability and reproducibility of the two methods of analysis, Lin’s concordance correlation coefficient (LCCC) was implied (freeware version 2020; https://www.statstodo.com/LinCCC.php) and results have been given in terms of accuracy. The mean (±SE), median, range, reference intervals (RIs) (25th to 95th percentile) and coefficient of variance (CV) were deduced for the overall as well as group-wise data keeping in view the guidelines provided by the American Society for Veterinary Clinical Pathology (ASVCP) [29] using the Reference Value Advisor (freeware v.2.1: http://www.biostat.envt.fr/reference-value-advisor).

## Results and discussion

The HAs are complex machines which are validated by the manufacturers, off-hand, before launching them in the market. These validations are carried out under ideal circumstances in concordance to the national/regional policies such as those provided by the FDA, USA and CE Mark, EU. However, the daily analytical and diagnostic performance of these HAs varies noticeably. In order to maintain valid and reliable results in daily testing, various international guidelines, such as those by ASVCP, Clinical Laboratory and Standards Institute (CLSI), International Organization for Standardization (ISO), have been provided which are upon the discretion of the clinical laboratories and laboratory personnel. These guidelines, apart from emphasizing on several verification items (precision, accuracy, linearity and comparability), also recommend performance evaluation of HAs. The present study is a novel one from Pakistan which addresses performance evaluation of Rayto RT-7600Vet–a multi-species HA- in comparison to manual hematological methods taking into account four main cbc attributes (TEC, Hb, PCV and PLT) in perspective. As there is no such study regarding the clinical performance of the model of HA used in the present study, hence the comparison of our results has been made with results reported for HAs of different other makes and models.

The results regarding normality testing of the hematological attributes attained through Rayto RT-7600Vet and through manual methods in the present study are given in Table 1. All the studied hematological attributes attained through HA had weak to moderate skewness (positive and negative). However, regarding manual hematological method, moderate (TEC-M, PCV-M, MCH-M) to high (Hb-M, MCV-M, MCHC-M) skewness was noticed. Regarding the results of kurtosis, all attributes attained through manual methods had high positive, leptokurtic distribution (having many outliers) except for PLT-M and PCV-M. On the contrary, the HA-deduced attributes had platykurtic distribution (having less outliers) except for PLT. Comparing our results with prior studies, it is evident that the literature is rife with data indicating least skewness and kurtosis in the data attained both from human and veterinary HAs, as compared to manual hematological methods [30, 31]. Higher skewness and kurtosis in our results for HAs could be an inherent/manufacturing characteristic of the machine. Platykurtic and least CV% for PCV-M in present study is due to the reason that the gold-standard microcentrifuge method was used in the present study which is considered best manual method practice for validating the HAs even today [27, 32].

**Table 1:** Normality testing results for various hematological attributes attained through hematology analyzer, Rayto RT-7600Vet (A) and manual method (M) in Cholistani cattle (n = 134).

Attributes	Skewness		Kurtosis		CV (%)
	Value	Interpret.	Value	Interpret.	
**HEMATOLOGY ANALYZER**					
Total erythrocytic count-A	-0.52	MN	0.75	LP, Platykurtic	20
Hemoglobin-A	-0.15	WN	-0.12	LN, Platykurtic	23
Packed cell volume-A	-0.77	MN	0.44	LP, Platykurtic	21
Mean corpuscular volume-A	0.28	WP	-1.28	HN, Platykurtic	16
Mean corpuscular hemoglobin-A	-0.098	WN	-1.18	HN, Platykurtic	16
Mean corpuscular hemoglobin concentration-A	0.85	MP	3.92	HP, Platykurtic	13
Platelet count-A	0.74	MP	1.24	HP, Leptokurtic	24
**MANUAL METHOD**					
Total erythrocytic count-M	0.65	MP	1.41	HP, Leptokurtic	30
Hemoglobin-M	1.11	HP	2.67	HP, Leptokurtic	21
Packed cell volume-M	0.64	MP	1.72	HP, Platykratic	16
Mean corpuscular volume-M	2.1	HP	5.6	HP, Leptokurtic	44
Mean corpuscular hemoglobin-M	0.76	MP	2.14	HP, Leptokurtic	59
Mean corpuscular hemoglobin concentration-M	1.00	HP	3.91	HP, Leptokurtic	22
Platelet count-M	-0.35	MN	-0.88	LN, Platykurtic	35

MN = moderate negative, WN = weak negative, WP = weak positive, HP = high positive, MP = moderate positive, LP = low positive, LN = low negative, HN = high negative, HP = high positive, CV = coefficient of variation

The CV for attributes attained through Rayto RT-7600Vet HA in the present study ranged from 16–24% being highest (24%) for PLT-A. Whereas, for attributes attained through manual methods, the CV ranged from 16–59% being highest (59%) for MCH-M followed by that in MCV-M (44%) and PLT-M (35%). Lowest CV was noticed for PCV-M (16%). The range of CV% for HA in the present study is higher than that reported earlier both for veterinary and human HAs. A lower CV (1–9%) has been reported for human blood using Sysmex® HA [30]. Similarly, while assessing performance evaluation of an Austrian HA (V Sight HA, Menarini Pharma, Austria) for bovine blood, it was elucidated that the CV% verified for all erythrocytic attributes should not be ≤5% [33]. Even lower range of 1–3% has been reported while assessing a Coulter HA (Coulter Diff hematology analyzer, USA) [4]. On similar pattern, yet another study conducted on performance evaluation of a Coulter HA (Coulter Electronics, UK) for bovine and equine blood, a lower CV% of 0.7–3% has been reported with 5–7% for PLT [34]. The CV (24%) for PLT in the present study though is higher, yet for platelets, their shape and clumping characteristic blood of all species have always hindered their accurate counting by various cell counters and HAs [35]. Previous studies have also reported higher than normal CV% for PLT. A study conducted on assessing the clinical efficacy of a Swedish HA (CA530, Boule Medical, Stockholm, Sweden), it was reported that the CV for all hematological attributes attained through this machine were within acceptable range except for PLT of dogs, cats and horses [18]. Considering the fact that higher CV% indicates lesser equipment precision, the efficacy of the HA used in this study seems questionable. As per the CLSI and ASVCP guidelines, the manufacturers must present within-batch and within-run precision in terms of CV% [36, 37]. However, the HA used in the present study (Rayto RT-7600Vet) did not have any information regarding CV% on its instruction’s manual.

The overall mean (±SE) values and RIs for various hematological attributes attained through two methods are given in Table 2. All the attributes attained through Rayto RT-7600Vet HA in the present study had lower mean (±SE) values and lower RIs as compared to those published earlier for this cattle breed conducted through Sysmex HA (Sysmex K21, Kobe, Japan) [21]. However, they were within the physiological ranges reported elsewhere [28]. While using similar HA (Rayto RT-7600Vet), a study on Bali cattle from Indonesia [10] and another one on Egyptian cattle [38] have reported values higher than ours. However, values very close to ours have been reported for HB, PCV and TEC in a study conducted on same breed of cattle (Cholistani, n = 360) using same HA [3]. As the results of the HA under discussion (Rayto RT-7600Vet) seem same for same breed in various studies, hence it seems inevitable to elucidate that the difference in values could be due to different breed and different type of HA used in other studies. On the contrary, the mean (±SE) values and RIs attained through manual methods in the present study are close to various studies conducted earlier on zebu humped cattle [3, 21, 28]. However, Hb-M had a very wide range (94.7–153.7g/L) in the present study. The Hb-M was estimated using gold-standard cyanmethemoglobin method in the present study. It has been well established that erroneous pipetting or imperfect time of incubation for the study samples could result in higher values [39, 40].

**Table 2:** Overall mean (±SE), median, interquartile range, minimum, maximum, 25^th^ to 95^th^ percentile of reference interval (RI) and 95% confidence interval (CI) for various hematological attributes attained through Rayto RT-7600Vet hematology analyzer (A) and manual method (M) in Cholistani cattle (n = 134).

Attributes	Mean (±SE)	Median (IQR)	Range (Min-Max)	RI (25^th^ to 95^th^)	95% CI
**HEMATOLOGY ANALYZER**					
Total erythrocytic count-A (10^12^/μL)	6.4±0.1	6.5(1.6)	6.6(2.4–9.0)	5.6–8.7	6.0–6.7
Hemoglobin-A (g/L)	88.5±2.6	87.0(28.0)	99.0(37.0–136.0)	75.7–116.7	83.1–93.8
Packed cell volume-A (%)	27.6±0.8	28.5(7.5)	27.2(10.3–37.5)	24.3–36.5	26.0–29.3
Mean corpuscular volume-A (fL)	43.4±0.8	41.4(10.2)	20.5(33.8–54.3)	38.8–53.8	41.7–45.2
Mean corpuscular hemoglobin-A (pg)	13.9±0.3	14.4(4.0)	8.3(9.8–18.1)	11.8–17.2	13.3–14.5
Mean corpuscular hemoglobin concentration-A (g/L)	321.4±5.3	311.0(18.5)	176.0(277.0–453.0)	301.0–427.7	310.7–332.1
Platelet count-A (10^3^/μL)	296.7±9.0	297.5(86.2)	324.0(174.0–498.0)	252.0–449.5	278.5–314.8
**MANUAL METHOD**					
Total erythrocytic count-M (10^12^/μL)	6.3±0.2	6.2 (2.5)	10.1(2.4–12.6)	5.0–9.3	5.8–6.8
Hemoglobin-M (g/L)	107.6±3.1	106.2(19.6)	122.0(68.0–190.0)	94.7–153.7	101.4–113.8
Packed cell volume-M (%)	30.1±0.6	29.5(6.0)	25.0(19.0–44.0)	27.0–38.7	28.8–31.3
Mean corpuscular volume-M (fL)	51.8±2.7	47.0(17.6)	105.6(27.1–132.7)	39.9–108.6	46.2–57.3
Mean corpuscular hemoglobin-M (pg)	18.8±1.2	16.1(7.1)	45.6(8.3–53.9)	13.6–45.8	16.3–21.2
Mean corpuscular hemoglobin concentration-M (g/L)	361.7±10.2	361.8(102.3)	363.0(220.3–583.3)	312.5–479.8	341.2–382.1
Platelet count-M (10^3^/μL)	251.3±12.2	259.5(154.2)	375.0(32.0–407.0)	170.0–399.5	226.7–275.9

**Fig 1:**
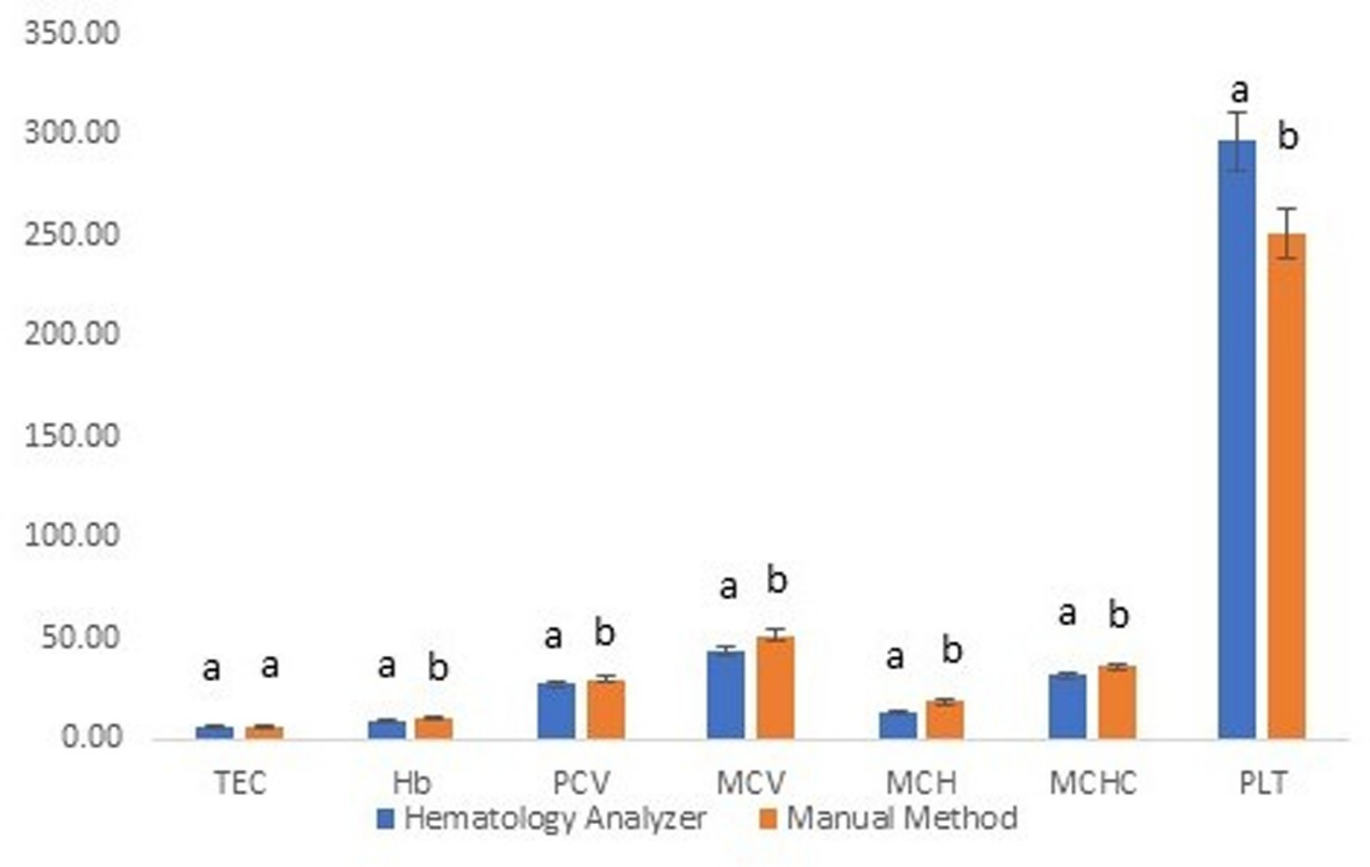
Overall results of various hematological attributes as deduced through hematology analyzer (Rayto RT-7600Vet) and manual hematological methods for Cholistani cattle blood (n = 134). Different superscripts on error bars differ significantly (P≤0.05) between the two methods for each hematological attribute.

Comparison between the overall results of hematological attributes attained through two methods in the present study revealed that all the studied attributes, except TEC, were significantly (P≤0.05) different for both methods (Fig 1). The Hb, MCV, MCH and MCHC were significantly (P≤0.05) higher for manual methods as compared to results of HA. The PLT was however, significantly (P≤0.05) lower (251.3±12.2×10^3^/μL) for manual methods as compared to that for HA (296.7±9.0×10^3^/μL). The results are in line with previous reports conducted on comparative efficacy of HAs in contrast to manual hematological methods. Hematological attributes (especially erythrocytic indices) have been reported to be higher through manual methods as compared to those attained through HA [41]. Similar results have also been reported for equine and bovine hemograms [42]. The inter-personal error could be attributed to this variability in results of manual methods as they are subjective in nature [43]. However, these manual methods are still in vogue for validating and checking the clinical/diagnostic efficacy of various automated equipment globally. In addition, it is noteworthy that platelet counting has always been spurious and erroneous even through HAs as reported earlier. In the impedance-based HAs, the platelets and RBCs, as particles, are suspended in an electrolyte solution and diluted suspension is passed through an aperture producing an impulse, which in turn is counted as a single cell. The platelet size and shape, allied with their clumping characteristic creates problems in their counting [25, 44].

**Fig 2:**
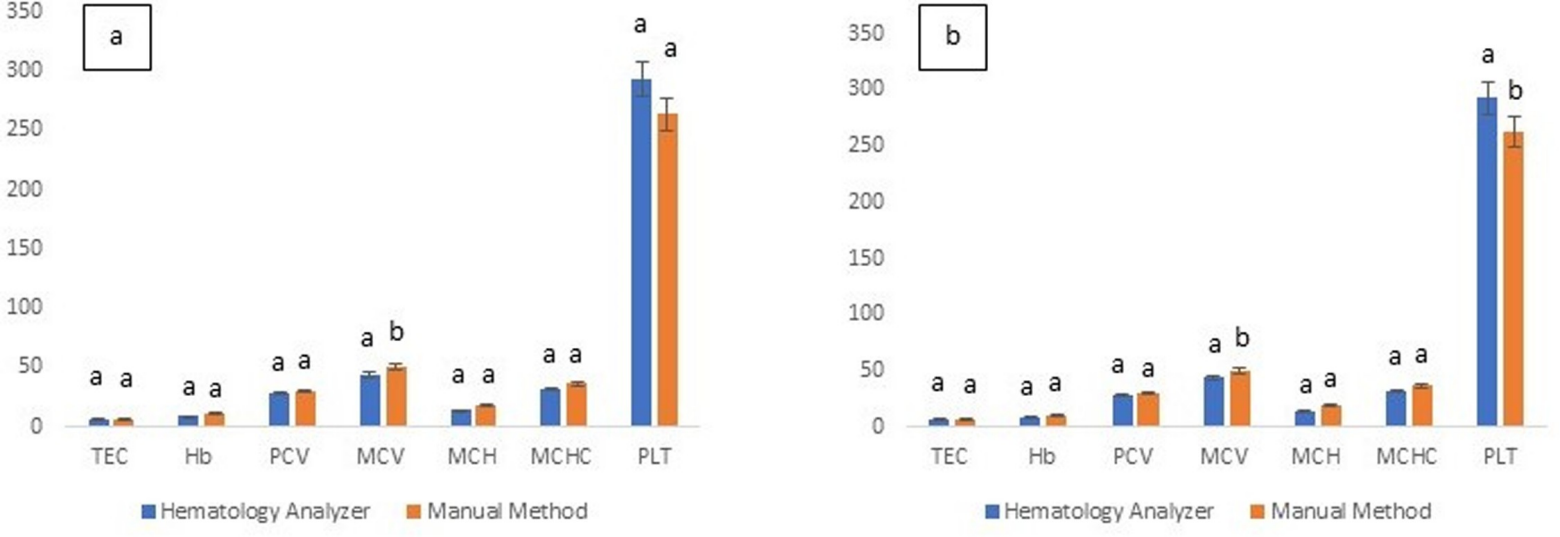
Gender-wise hematological attributes as deduced through hematology analyzer (Rayto RT-7600Vet) and manual hematological methods for Cholistani cattle blood a: in females (n = 96) and b: in males (n = 38) (b). Different superscripts on error bars differ significantly (P≤0.05) between the two methods for each hematological attribute.

**Fig 3:**
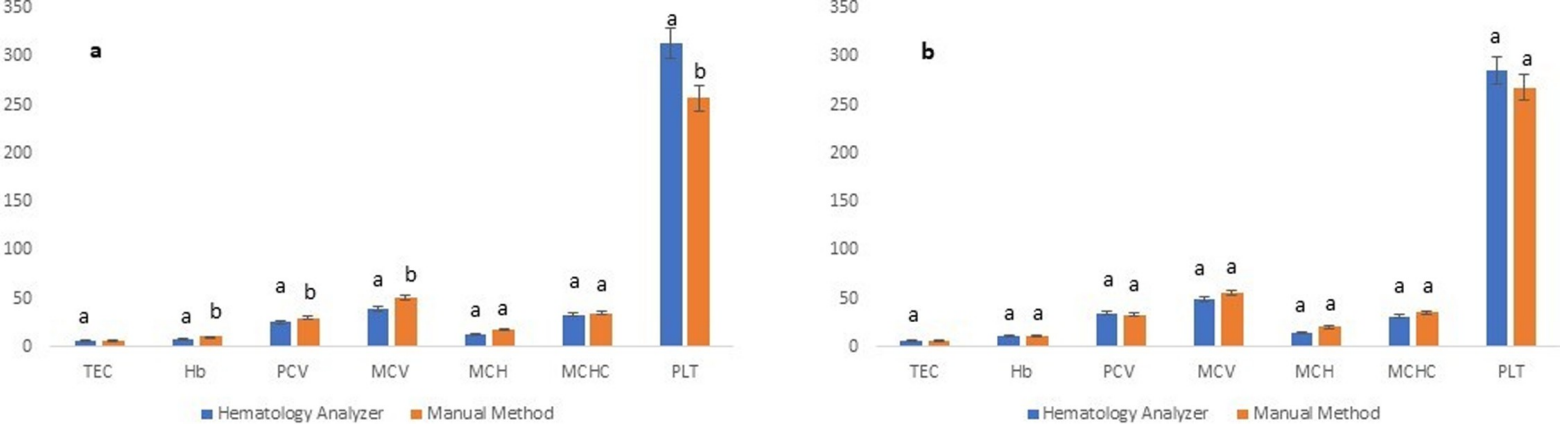
Age-wise hematological attributes as deduced through hematology analyzer (Rayto RT-7600Vet) and manual hematological methods for Cholistani cattle blood a: in young (n = 47) and b: in adults (n = 87). Different superscripts on error bars differ significantly (P≤0.05) between the two methods for each hematological attribute.

Gender-based results revealed that in the females, the TEC, Hb, PCV and PLT were not significantly (P≥0.05) different for the two analytical methods (Fig 2A). However, in males, PLT was significantly (P≤0.05) higher through HA as compared to manual methods (Fig 2B). Regarding the age-based groups of the present study, in the adult animals (n = 87), no significant (P≥0.05) difference was noticed between results of all the studied attributes attained through HA and manual methods (Fig 3A and 3B). Similar variability is results have been shown by earlier studies as well both while reporting performance of human HAs [45, 46] and veterinary HAs. As per the guidelines of International Committee for Standardization of Hematology (ICSH) and ASVCP, at least 120 healthy individuals should be incorporated in a study directed towards ascertaining RIs of a population. Furthermore, the guidelines also indicate that separate RIs may be developed for different cohorts/groups such as those based upon age, gender or any other categorical factor [24, 32, 37].

**Fig 4:**
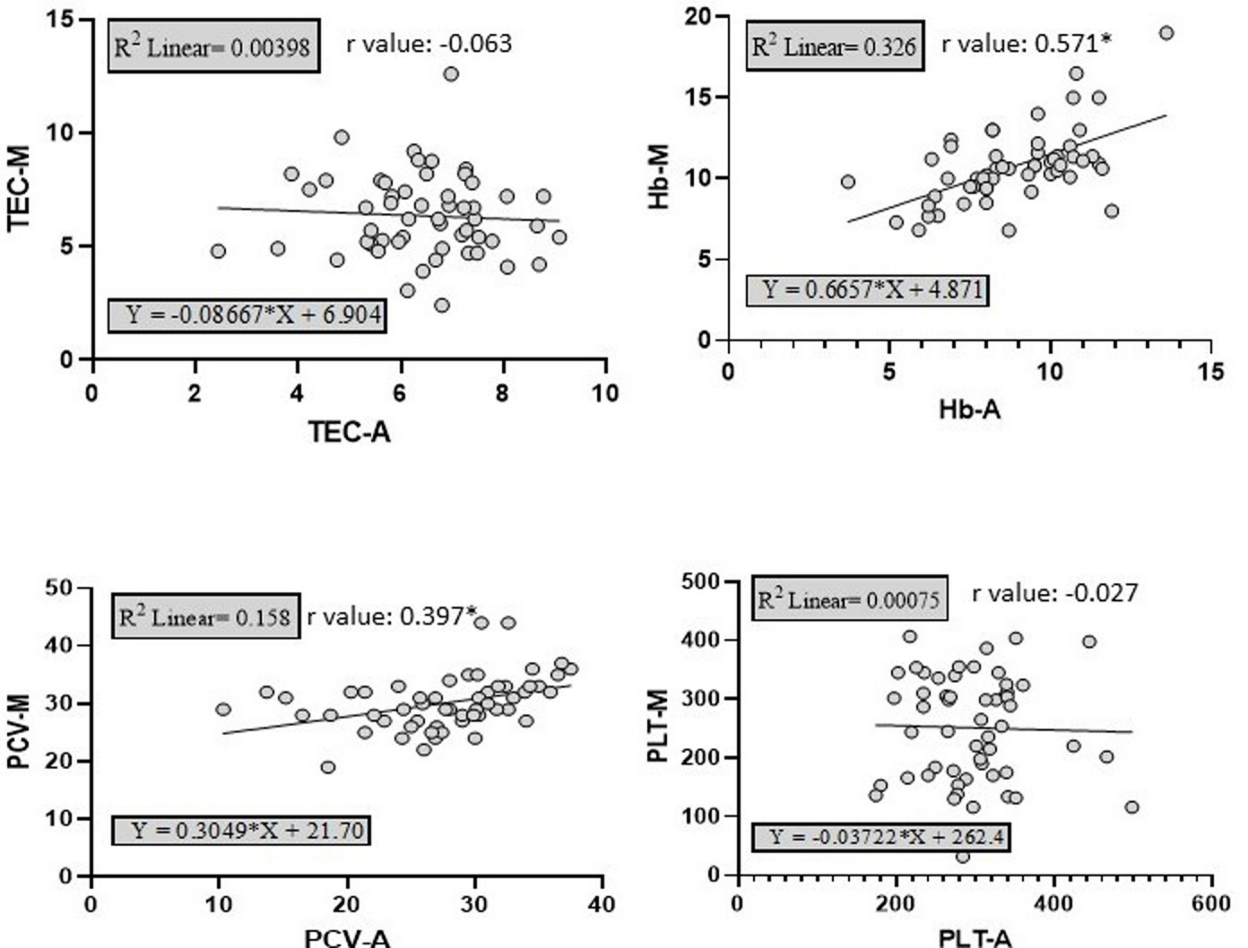
Logilinear regression between various hematological attributes as deduced through hematology analyzer (Rayto RT-7600Vet) (TEC-A, HbA, PCV-A and PLT-A) and manual hematological methods (TEC-M, Hb-M, PCV-M and PLT-M) for Cholistani cattle blood (n = 134).

The results regarding interrelationship between the four main hematological attributes (TEC, Hb, PCV, PLT) attained through two methods of the present study (HA *vs* manual methods) have been given in Fig 4. A weak relationship was noticed between the attributes attained through HA and those through manual methods as indicated by weak r-values and adjusted r-square values. Highest relation was revealed only between Hb-A and Hb-M (r-value = 0.571; r-square value = 0.326; adj. r-square = 0.314 i.e. 31.4% probability). Our values are far lower than those reported earlier between attributes attained by the manual methods and through HAs both for human and veterinary medical practice which ranged from 0.897 to 0.941 [43, 47]. Higher correlations have been reported for goat blood while assessing the HA (Adiva, Siemens, Germany) against manual hematological methods [48].

**Fig 5:**
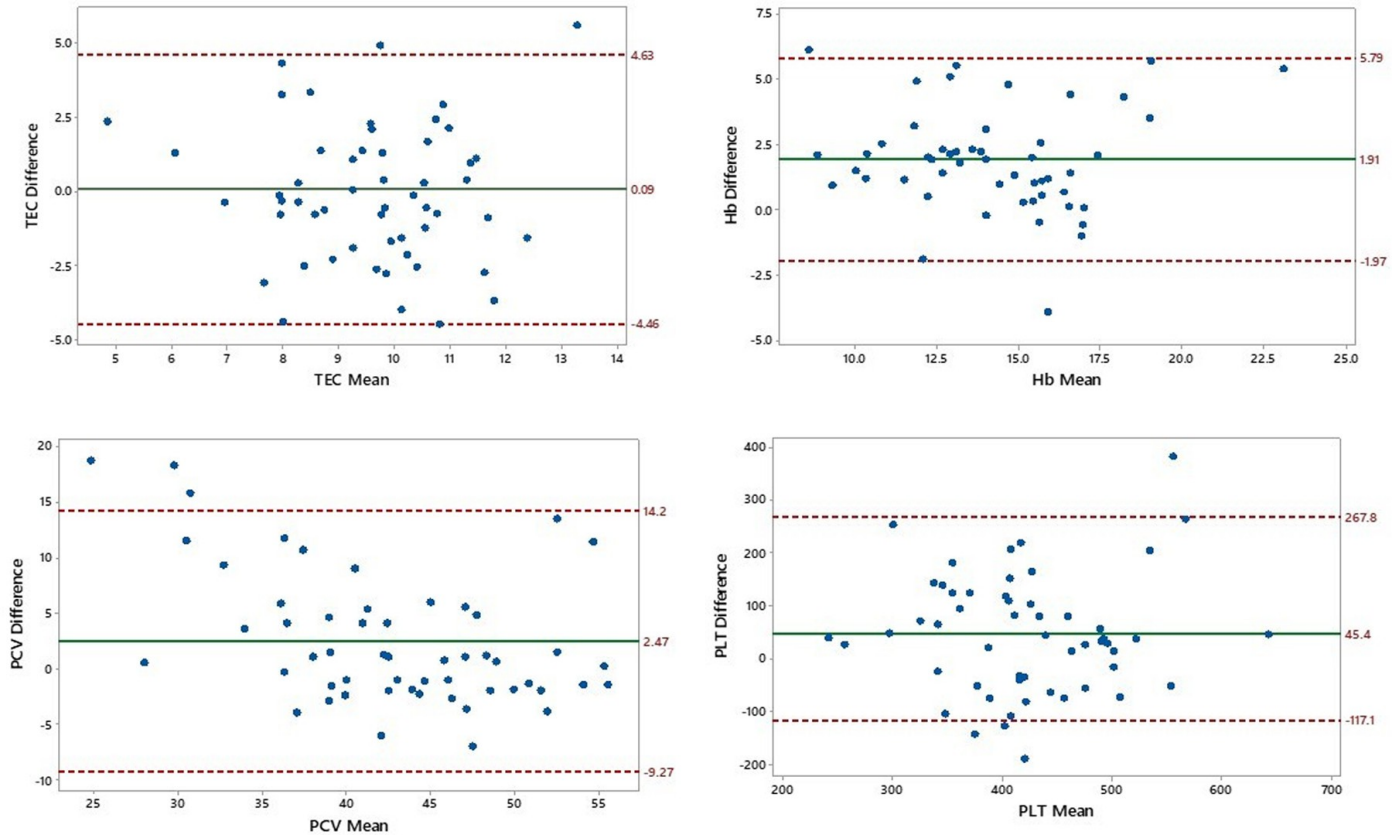
Scatterplot of Bland and Altman test between the difference and mean of various hematological attributes attained through hematology analyzer (Rayto RT-7600Vet) and manual hematological methods for Cholistani cattle blood (n = 134). Solid green line indicates mean difference whereas the upper and lower red dotted lines indicate upper and lower values for 95% CI, respectively.

For the present study, three tests for assessing the level of agreement between the two methods of hematological analysis were implied *i*.*e*. Bland and Altman (Fig 5), Cronbach Alpha and ICC, apart from the LCCC test for measuring accuracy (Table 3). The reliability level of estimating Hb and PCV had highest ICCC value of 0.722 and 0.555 for average measures. However, accuracy level, as determined through LCCC, was highest for TEC (0.9504) followed by that in PCV (0.8743), PLT (0.8202) and Hb (0.7041), respectively for both analytical methods. Poor level of agreement, in general, was shown for the two methods of analysis regarding all four hematological attributes (Fig 5). Only Hb methods of estimation had closer agreement (upper 95% CI = 5.79; lower 95% CI = -1.97; SD of bias = 11.24). Previous research works have not implied these tests of agreement between manual and HA-deduced hematological attributes, hence, cannot be discussed in specific. However, the Bland and Altman test have been extensively implied while conducting a comparative performance evaluation of two HAs [49–51].

**Table 3:** Cronbach alpha, intraclass correlation, Lin’s concordance coefficient and mean bias error (MBE) between various hematological attributes attained through hematology analyzer (A) and manual method (M) in Cholistani cattle (n = 134).

Intraclass Correlation		95% CI	Cronbach Alpha	Accuracy	MBE
**TEC-A versus TEC-M**					
Single Measure	-0.060	-0.321–0.209	-0.128	0.9504	-0.087
Average Measures	-0.128	-0.944–0.346			
**Hb-A versus Hb-M**					
Single Measure	0.565	0.352–0.722	0.722	0.7041	1.912
Average Measures	0.722	0.521–0.839			
**PCV-A versus PCV-M**					
Single Measure	0.384	0.132–0.589	0.555	0.8743	2.47
Average Measures	0.555	0.233–0.742			
**PLT-A versus PLT-M**					
Single Measure	-0.026	-0.290–0.241	-0.054	0.8202	-45.0
Average Measures	-0.054	-0.816–0.389			

Keeping in perspective the results of the present study, it is concluded that the HAs (specifically Rayto RT-7600Vet, China) may present data having higher skewness, kurtosis, and CV%, however, they are valid for multi-species hematological analysis. The results of this study could be a benchmark for laboratories/clinical settings (especially of resource-poor settings) which are using multi-species veterinary HAs. Caution must however, be taken in interpreting their results. It is recommended that each laboratory/clinical setting(s) may devise specifically-corrected RIs and CV% for their analyzers regarding each tested hematological attribute. Periodic verification protocol for HAs may also be devised. Accuracy and precision studies for multi-species veterinary HAs, with higher population and with blood of different species, need attention. Furthermore, PLT count needs further validation through manual counting methods. On grounds similar to those of this study, the white blood cell counts may also be validated for these machines in comparison to manual methods.
